# Influence of Platelet-Rich Plasma on Recurrent Vesicovaginal Fistula—A Histological and Immunohistochemical Study

**DOI:** 10.3390/jcm13020370

**Published:** 2024-01-10

**Authors:** Dominika Ewelina Streit-Ciećkiewicz, Justyna Szumiło, Magdalena Emilia Grzybowska, Konrad Futyma

**Affiliations:** 1 2nd Department of Gynecology, Medical University in Lublin, Jaczewskiego 8, 20-954 Lublin, Poland; 2 Department of Clinical Pathomorphology, Medical University in Lublin, Jaczewskiego 8b, 20-090 Lublin, Poland; justynaszumilo@umlub.pl; 3 Department of Gynecology, Obstetrics and Neonatology, Medical University of Gdańsk, Ul. Smoluchowskiego 17, 80-214 Gdańsk, Poland; mgrzybowska@gumed.edu.pl

**Keywords:** vesicovaginal fistula, immunohistochemistry, histology, platelet-rich plasma, growth factors

## Abstract

Vesicovaginal fistula is a cause of deterioration in the quality of life. It is a communication between the bladder and vagina resulting in the uncontrollable leakage of urine through the vagina. Recently, regenerative methods have been used more frequently, and platelet-rich plasma is one of these methods. The functional properties of platelet-rich plasma are based on the synthesis and secretion of multiple growth factors released after platelet activation. The aim of this study was to investigate how platelet-rich plasma influences the condition of the tissue and the healing ability of the urothelium, vaginal epithelium and tissues surrounding the fistulous canal. The study included eight patients who had undergone the Latzko procedure aimed at closing the vesicovaginal fistula. Samples were collected during primary surgery without platelet-rich plasma and after failed surgery, during a second attempt. The specimens were subjected to histological and immunohistochemical evaluation. The histology demonstrated that in platelet-rich plasma patients, greater vascularization and wider subepithelial mucosa layering was observed. Inflammatory infiltration in the subepithelial layer was increased in platelet-rich plasma patients. No localization differences in growth factor proteins were found in either group, but in platelet-rich plasma-patients, the reactions were stronger. It can be concluded that the use of platelet-rich plasma improves the morphological structure of the injected tissues.

## 1. Introduction

Vesicovaginal fistula (VVF) is a cause of significant deterioration in the quality of life. It is a non-physiological communication between the bladder and the vagina and results in an uncontrollable, often constant, leakage of urine through the vagina [1]. Surgical treatment still remains the main treatment option and, according to the WHO, the successful closure rate for first repairs is around 85% [2]. Surgical treatment can also be performed via the transabdominal approach with a laparotomy or laparoscopy, or even robotically. Recently, regenerative methods have been used more frequently, and more and more reports are published proving their supporting role in standard treatment. 

Platelet-rich plasma (PRP) is one of these regenerative methods. PRP is an autologous platelet preparation obtained from whole blood, with a 3–18 times higher concentration of thrombocytes than in the starting preparation. Generally, the functional properties of PRP are mainly based on the synthesis and secretion of multiple growth factors that are exuded after platelet activation, which explains their role in wound healing processes [3,4]. Activated platelets change their shape, can move inside the matrix and regenerate new tissue more effectively. The released growth factors are ligands that interact with the appropriate receptors, and growth factors regulate the expression of other factors and are inhibited in negative feedback mechanisms [4,5,6].

Platelet derived growth factor (PDGF) is among the most important PRP growth factors—it is composed of A and/or B subunits, and three isoforms exist: AA, BB, and AB. PDGF stimulates chemotaxis, mitosis of fibroblasts, the synthesis of collagen and the secretion of TGFβ from macrophages [7]. Transforming growth factor-β (TGFβ), which stimulates the production of collagen, prevents collagen breakdown, promotes angiogenesis, connective tissue regeneration and chemotaxis of the immune cells, and is also one of the major growth factors of platelet granules [8]. Vascular endothelial growth factor (VEGF) stimulates vessel formation and, therefore, brings nutrients and increases blood flow at the site of injury [9]. Epidermal growth factor (EGF) stimulates chemotaxis and angiogenesis of endothelial cells and the mitosis of mesenchymal cells. Different studies have shown that EGF promotes epithelization and markedly shortens the healing process [7,10]. Fibroblast growth factor (FGF) represents one of the most potent mitogens and demonstrates manifold actions on multiple cell types. Together with VEGF, FGF is involved in the process of angiogenesis [7]. The most important growth factors (GF) are presented in Table 1.

Platelets begin to release growth factors during the initial 10 min following the start of coagulation processes. The level of growth factors released is correlated with the activated thrombocytes, the method of blood collection, the speed, time and temperature of centrifugation, the overall preparation time and the activating agents added to the PRP. There are many commercial systems that create PRP from autologous whole blood, however, there is still no unique protocol for PRP production [11].

Despite PRP implementation in many different urogynecological indications, little is known of what real impact it has on tissue histological rearrangement and how this rearrangement influences wound healing, especially in case of wounds exposed to different discharges and excretions.

## 2. Materials and Methods

The study was performed in the II Department of Gynecology of the Medical University in Lublin and included 46 patients—24 in the research group injected with PRP and 22 in the control group without PRP. Each patient had undergone the Latzko procedure aiming to close the VVF. Specimens of tissues surrounding fistula canal were collected from 8 patients for immunohistochemical and histological tests. The Local Ethics Committee approved the study concept (KE-0254/363/2018). The primary Latzko was performed was performed at least 3 months after the occurrence of a fistula following hysterectomy. If this procedure was unsuccessful, patients were injected with PRP after an additional 3 months in order to allow for local inflammation resolution in fistulous tissues. A secondary Latzko procedure was performed 6 weeks after the PRP injection.

In order to obtain PRP, 20 mL of whole venous blood was collected from each patient with a syringe containing 2 mL of an anticoagulant (sodium citrate). All samples were prepared with the A-PRP Novareg kit (Novareg sp z o.o. Kielce, Poland). Then, the specimens were centrifuged for 8 min at 1900 rpm and the whole blood was separated into different fractions containing appropriate ingredients. Five milliliters of PRP was then collected into a separate syringe and the patient was injected within 10 min after centrifugation. With the patient in the lithotomy position, the exact location of the fistula was determined and the 5 mL of PRP was injected transvaginaly into 4 to 5 points around the fistulous canal vaginal orifice with Secalon-T™ 130 mm needle (Merit Medical, South Jordan, UT, USA).

Samples of fistulous canals were collected during primary surgery without PRP and if surgery was unsuccessful, again during the second attempt. The Latzko procedure was performed 6 weeks after PRP injection. Specimens were subjected to histological and immunohistochemical evaluation. Microscopic evaluation of the tissues included comparison of vascularity, collagen fibers and inflammatory infiltration in patients with or without PRP injection.

Additionally, immunohistochemical evaluation was carried out in order to analyze the presence of the proteins that are the most important in wound healing: platelet derived growth factor (PDGF), epithelial growth factor (EGF) and transforming growth factor (TGF). Tissues were fixed in a 10% buffered formalin solution and then transferred to paraffin blocks. Hematoxylin and eosin (HE) staining, van Gieson and Gomori’s silver impregnation were applied. Immunohistochemical tests were performed on the paraffin blocks, from which 4 mm thick sections were cut and placed on salinized slides. The PTLink device (Agilent Technologies, Agilent Dako, Santa Clara, CA, USA) was used to remove the paraffin and exposed the antigens. The blocks were then placed for 20 min at 97 °C in Target Retrieval Solution (pH = 9.0 for PDGFB, TGFα, TGFβ, EGF and pH = 6.0 for PDGFA) (Agilent Technologies, Agilent Dako, Santa Clara, CA, USA). Endogenous peroxidase activity was blocked by covering sections with 3% hydrogen peroxide solution for 5 min at room temperature. Subsequently, specimens were incubated with primary antibodies (PDGFAA-07-1436, Merck, Sigma-Aldrich, Darmstadt, Germany; PDGFB-SAB2108198-100UL954, Merck, Sigma-Aldrich, Darmstadt, Germany; anti-TGF β1-SAB4502954, Merck, Sigma-Aldrich, Darmstadt, Germany; anti-TGF β2-SAB4502956, Merck, Sigma-Aldrich, Darmstadt, Germany; anti-EGF SAB5300488, Merck, Sigma-Aldrich, Darmstadt, Germany) and covered with Dako Real Envision (Dako K5007; HRP, Rabbit/Mouse, Agilent Technologies, Agilent Dako, Santa Clara, CA, USA) for 30 min at room temperature. After each stage of reaction, the specimens were washed by Dako Wash Buffer (Agilent Technologies, Agilent Dako, Santa Clara, CA, USA).

In order to visualize the reaction in a light microscope, a 3,3′- diaminobenzidine tetrahydrochloride solution was applied. Next, the specimens were stained with Mayer’s hematoxylin for 1 min (Merck, Sigma-Aldrich, Darmstadt, Germany). Rabbit serum was employed as a negative control. Positive control was performed on scraps from the human kidney and placenta. Reactions were evaluated in an Olympus BX51 light microscope (Olympus, Tokio, Japan). Patients’ demographic and clinical data are given in Table 2.

## 3. Results

**Figure 1:**
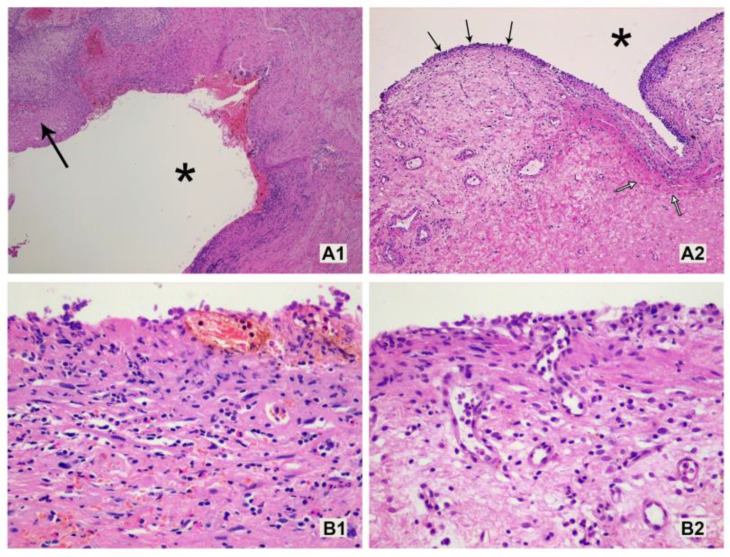
Microscopic appearance of vesicovaginal fistula without (**A1**,**B1**) and after (**A2**,**B2**) PRP injection. After injection, inflammatory infiltrate composed mostly of lymphocytes and plasma cells as well as the proliferation of new capillaries and more pronounced fibrosis in the subepithelial layer were seen (asterisk—lumen of the fistula, thick black arrow—squamous epithelium of the vagina, thin black arrow—thin epithelium partly covering the fistula channel, white arrow—subepithelial fibrosis). (HE; (**A1**,**A2**)—objective magnification ×5; (**B1**,**B2**)—objective magnification ×20).

Histological examination demonstrated some slight differences in the wall of the vesicovaginal fistula without and after PRP injection (Figure 1). In samples from patients after injection, greater vascularization and more pronounced subepithelial fibrosis were seen. Moreover, inflammatory infiltration in the subepithelial layer was markedly increased in PRP patients and consisted mostly of lymphocytes and plasma cells when compared to non-PRP patients with more numerous eosinophils. We also assumed that GFs would be exerted on all components of injected fistulous tissues. Unfortunately, we did not observe any change in collagen fibers before and after PRP injection in the investigated samples.

**Figure 2:**
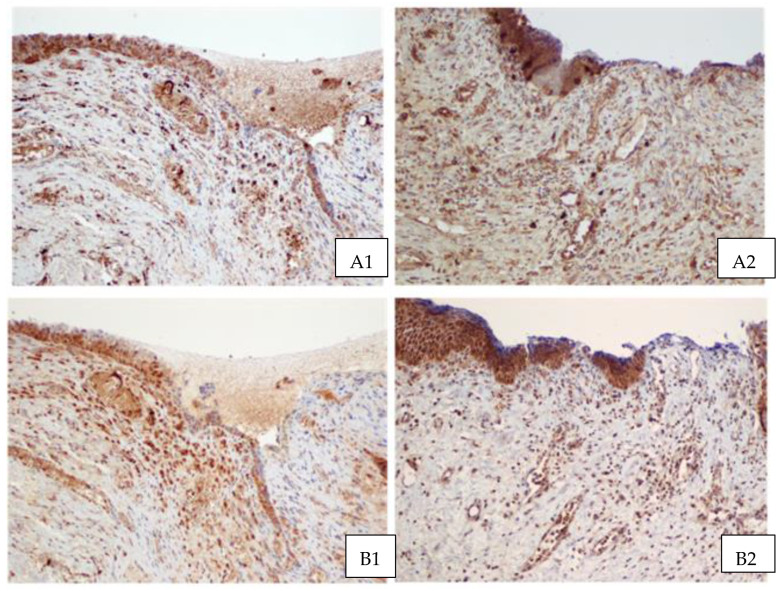

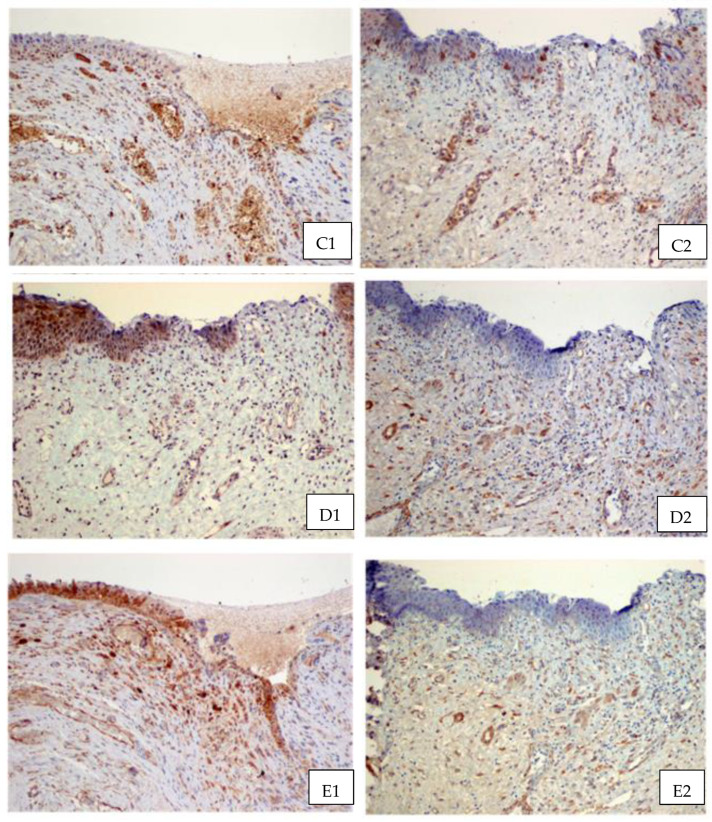
Comparison of immunohistochemical reactions in the wall of vesicovaginal fistulas without (on the left) and after PRP injection (on the right): EGF (**A1**,**A2**), TGF-β1 (**B1**,**B2**), TGF-β2 (**C1**,**C2**), PDGF AA (**D1**,**D2**) and PDGFβ (**E1**,**E2**). (EnVision/HRP; objective magnification ×10).

In the immunohistochemistry analysis (Figure 2), EGF presented mainly a localized cytoplasmic reaction in the stratified flat epithelium, fistula epithelium and macrophages. In the case of TGF-β1, a membrane-cytoplasmic reaction localized in the fistula epithelium, smooth muscles cells of blood vessels, as well as in fibroblasts and macrophages was evident. In contrast, TGF-β2 presented a nuclear reaction in the fistula epithelium, single vascular epithelial cells, fibroblasts and macrophages, and a cytoplasmic reaction in blood vessel smooth muscle cells and the vaginal epithelium. The presence of PDGFA was observed in the membrane–cytoplasmic reaction and it was identified in the smooth muscles cells of blood vessels, vaginal epithelium and macrophages.

Immunohistology staining with PDGFB was similar to the PDGFA membrane–cytoplasmic reaction, and was localized in the vessel epithelium, blood vessel smooth muscle cells, vaginal epithelium and macrophages. We saw no differences in the localization of GF proteins between PRP and non-PRP tissues, but in PRP patients, the reactions were stronger and immunohistochemical reactions were more expressive. The reaction types of this protein in each tissue sample are presented in Table 3.

## 4. Discussion

In recent years, VVF treatment, especially recurrent VVF, has been considered to be required due to impaired healing processes with regard to bladder wall damage. Therefore, the use of supportive techniques that enhance healing appears to be an integral part of the therapeutic process. Platelet-rich plasma seems to be one of the best methods to apply because of its simplicity, cost-effectiveness and patient safety. Platelet granules contain GF’s that enhance healing, the expansion of the blood vessels and the reconstruction of the extracellular matrix.

In our study, histological evaluation revealed no significant differences between PRP and non-PRP samples, apart from increased amounts of blood vessels and a thicker subepithelial tissue layer in PRP patients. Immunohistochemical reactions were similarly located in both patient groups and no significant differences were observed between them, except with regard to TGF-β1 (where cytoplasmic reactions were observed in the fistula epithelium and blood vessel smooth muscle cells), and to TGF-β2 (which presented a nuclear reaction in the fistula epithelium, single vascular epithelial cells, fibroblasts and macrophages, and a cytoplasmic reaction in blood vessel smooth muscle cells and the vaginal epithelium).

Significant differences were observed in tissue infiltration density with different types of white blood cells between non-PRP and PRP patients. In the case of non-PRP patients, eosinophils were numerous, whereas in PRP patients, lymphocytes were predominant. Eosinophils participate in fibrin formation through the local secretion of tissue factor (TF) and thrombin. Additionally, they participate in plasminogen production that enhances the adhesive properties of eosinophils which, in turn, contributes to fibrinolysis processes. Moreover, as a result of degranulation, eosinophils release proteins, thus inducing pathways leading to tissue remodeling processes [12,13,14,15,16,17].

The lymphocyte predomination in the case of the PRP group could be explained by the induction of healing processes by factors released from platelet granules. As a result of platelet degranulation, damage association molecular patterns (DAMPs) are released and monocytes and neutrophils are activated. Recent studies have shown that the antimicrobial neutrophil peptide cathelicidin can promote T cell differentiation towards Th17 cells, which enhances wound healing through bacteria elimination, immune system response modulation and tissue remodeling [18,19].

In the literature, the only available VVF tissue immunohistological analysis was that which was undertaken by Medvedev et al. [20]. Therein, they assessed the morphological structure of VVF tissues before and after PRP injection, and their histological examinations showed a reduction in inflammation process activity, a complete resolution of erosive and ulcerative lesions and a reduction of the fibrosis degree of the stromal layer in their PRP group.

In the study published by Pengcheng Xu et al. [21], the role of PRP in wound healing in a mouse model was investigated. They showed that wound healing in the PRP group was significantly accelerated compared with the control group. In their study, they also assessed CD31 expression (the marker of neovascularization and angiogenesis) and demonstrated its overexpression in their PRP group. They also found that PRP treatment enhanced VEGF production in wound tissues, which also had a positive impact on angiogenesis. Moreover, they confirmed the positive effect of PRP on collagen formation and re-epithelialization.

Nolan et al. [22] carried out a histological analysis of fat grafting with concomitant PRP injection in diabetic foot ulcers. This was a three-armed study comparing patients with fat grafting, fat grafting with PRP and routine care for the treatment of diabetic foot ulcers in individuals who had undergone wound biopsies. The study confirmed that the application of fat with PRP in the case of diabetic foot ulcers intensified angiogenesis and provided better fat graft survival, as demonstrated by an increased density of adipocytes seen in wound biopsies.

Only a few studies exist in the current literature of the effects of PRP on the histology and immunohistochemistry of injected tissues, especially in human models, but those that are available are encouraging and subsequently promote the conception of novel treatment in many different fields of medicine.

## 5. Conclusions

The use of platelet-rich plasma significantly improves the morphological structure of injected tissues. Increased amounts of blood vessels and thicker subepithelial tissue layers in PRP patients were observed. The faster and more accurate healing processes in PRP patients could be explained by predominant lymphocyte infiltration followed by monocyte and neutrophil activation resulting in a higher antimicrobial potential and immune system response modulation in healing tissues. Moreover, the thickened collagen fiber network that is rebuilt during the healing process significantly increases the probability of fast and effective scar formation and the closure of the VVF. In addition, the cellular infiltration of PRP patient tissues differs from that of non-PRP patients, and is the result of immunological pathway activation and the mechanism of the initiation of the healing process.

## Figures and Tables

**Table 1:** Growth factors and their biological functions.

Name of GF	Abbreviation	Function
Platelet Derived Growth Factor	PDGF	Enhances collagen synthesis, fibroblast chemotaxis and macrophage activation
Transforming Growth Factor β	TGF β	Enhances synthesis collagen type I, promotes angiogenesis
Vascular Endothelial Growth Factor	VEGF	Stimulates angiogenesis, migration and mitosis of endothelial cells, as well as chemotaxis of macrophages, increases permeability of the vessels
Fibroblast Growth Factor	FGF	Promotes proliferation of mesenchymal cells
Epidermal Growth Factor	EGF	Stimulates cellular proliferation and differentiation of epithelial cells

**Table 2:** Patients’ demographic and clinical data.

Number of the Sample	Age	Parity	BMI	Surgery Before VVF Occurrence
0.01	49	1	24.2	Op. m. Meigs
0.0.1.1	49	1	24.2	Op. m. Meigs
0.02	54	1	30	TAH/BSO
0.03	42	0	19.53	TAH
0.04	33	1	20.2	Op. m. Meigs
0.05	74	1	19.71	TAH
0.06	60	4	26.7	TAH/BSO
0.07	44	0	23.5	TAH/BSO

Legend: Op. m. Meigs: Wertheim—Meigs radical hysterectomy; TAH/BSO: total abdominal hysterectomy with bilateral salpingo-oophorectomy; TAH: total abdominal hysterectomy.

**Table 3:** Types of growth factor reactions.

	Type of Reaction		
Growth Factors	Cytoplasmic	Nuclear	Membrane–Cytoplasmic
EGF	+		
TGFβ1			+
TGFβ2	+	+	
PDGFAA			+
PDGFB			+

## Data Availability

The data presented in this study are available on request from the corresponding author.
